# A comparison of four clustering methods for brain expression microarray data

**DOI:** 10.1186/1471-2105-9-490

**Published:** 2008-11-25

**Authors:** Alexander L Richards, Peter Holmans, Michael C O'Donovan, Michael J Owen, Lesley Jones

**Affiliations:** 1 Department of Psychological Medicine, School of Medicine, University Hospital Wales, Heath Park, Cardiff, Wales, UK, CF14 4XN

## Abstract

**Background:**

DNA microarrays, which determine the expression levels of tens of thousands of genes from a sample, are an important research tool. However, the volume of data they produce can be an obstacle to interpretation of the results. Clustering the genes on the basis of similarity of their expression profiles can simplify the data, and potentially provides an important source of biological inference, but these methods have not been tested systematically on datasets from complex human tissues. In this paper, four clustering methods, CRC, k-means, ISA and memISA, are used upon three brain expression datasets. The results are compared on speed, gene coverage and GO enrichment. The effects of combining the clusters produced by each method are also assessed.

**Results:**

k-means outperforms the other methods, with 100% gene coverage and GO enrichments only slightly exceeded by memISA and ISA. Those two methods produce greater GO enrichments on the datasets used, but at the cost of much lower gene coverage, fewer clusters produced, and speed. The clusters they find are largely different to those produced by k-means. Combining clusters produced by k-means and memISA or ISA leads to increased GO enrichment and number of clusters produced (compared to k-means alone), without negatively impacting gene coverage. memISA can also find potentially disease-related clusters. In two independent dorsolateral prefrontal cortex datasets, it finds three overlapping clusters that are either enriched for genes associated with schizophrenia, genes differentially expressed in schizophrenia, or both. Two of these clusters are enriched for genes of the MAP kinase pathway, suggesting a possible role for this pathway in the aetiology of schizophrenia.

**Conclusion:**

Considered alone, k-means clustering is the most effective of the four methods on typical microarray brain expression datasets. However, memISA and ISA can add extra high-quality clusters to the set produced by k-means, so combining these three methods is the method of choice.

## Background

Clustering genes according to their expression profiles is an important step in interpreting data from microarray studies. Clustering can help summarise datasets, reducing tens of thousands of genes to a much smaller number of clusters. It can aid understanding of systemic effects; looking for a small change in expression between disease states across many genes in a cluster could be a better strategy for finding the causes of complex, polygenic disorders than looking for large changes in single genes[1]. Clustering can also help predict gene function, as coexpressed genes are more likely to have similar functions than non-coexpressed genes[2].

There are many clustering methods for microarray expression data currently available[3]. However, there are few comparisons of these methods, making it hard for researchers to make a rational choice between them. The majority of papers comparing multiple clustering methods use simulated data or data from simple organisms such as bacteria and yeast [4-6], which may limit the applicability of their findings to data from more complex sources such as human tissues which express more genes. Thus, to investigate human disease, it would be useful to test the methods upon expression data derived from complex human tissues, among which brain tissue is particularly complex since it expresses a higher proportion of the genome transcribed than other tissues[7,8]. Thalamuthu *et al *[9] have previously looked at a wide range of datasets, including some human expression datasets. However, since they restricted their analysis to functionally defined subsets of genes, that analysis did not fully reflect the complexity of human expression, particularly for disorders where there is insufficient knowledge of their aetiology to focus on specific subsets of genes.

We have examined four methods, k-means clustering[10], Chinese Restaurant Clustering (CRC)[11], the Iterative Signature Algorithm (ISA)[12,13] and a new, progressive variant of ISA called memISA. memISA was loosely based upon another method called PISA, for which there was no suitable implementation[14]. These were chosen after a literature survey of the available methods (see table in Additional Files 1). All four are unsupervised methods that derive the clusters from the input data, rather than supervised methods which classify genes into user-specified clusters.

Many of the available comparative clustering studies focus exclusively on older methods [5,15], or restrict their analysis to a single class of clustering methods [4,6]. In our study, the methods were chosen on the basis of variety. ISA and memISA are examples of biclustering methods, CRC is a mixture model based method, while k-means clustering is a simple, well-understood algorithm. They were reported as performing well by their authors and/or other studies [4,5].

The methods were also chosen partly on the basis of novelty. Apart from k-means clustering, they are too recent to have been included in many previous surveys of clustering methods, and so are particularly in need of testing.

We compared the performance of these three methods by examining the results for biologically meaningful clustering by looking for gene ontology (GO) enrichments within the resulting clusters. We also generated and compared a modified variation of ISA, memISA, which weighted against genes that were already members of a cluster to prevent bias of clusters detected from the strongest genes within them.

## Methods

### Datasets

Three datasets were used for testing, the Dobrin [16], McLean 66 [17] (MC66) and Perrone-Bizzozero (PB – GEO dataset GSE4036) [18] datasets (Table 1). They were downloaded in CEL format from the Stanley Medical Research Online Genomics database[16], the Harvard National Brain Databank database[17] and GEO[19], respectively. They were then processed using R[20], with custom CDF files to map the probes to genes[21]. Box plots were used to examine the quality of the data, and several outlier samples were removed. Three versions of each dataset were produced. One was normalised by the RMA median polish method, for use in CRC and k-means[22]. The other two were normalised to produce a gene-normalised and sample-normalised dataset for running ISA[12].

**Table 1:** Datasets used to test clustering methods

	Pre quality control number of samples			Post quality control number of samples			Tissue	Chip	Number of genes
				
	Control	SCZ	BP	Control	SCZ	BP			
Dobrin	25	26	27	20	22	22	Brodmann Area 46	Affymetrix 133 plus 2.0	20292

McLean 66	27	18	19	27	15	19	Dorsolateral Prefrontal Cortex	Affymetrix 133A	12757

Perrone-Bizzozero cerebellum	14	14	0	14	14	0	Cerebellum	Affymetrix 133 plus 2.0	20292

Quality control consisted of box plotting the samples and removing outliers.

### Gene coverage

Gene coverage, the percentage of genes on the chip that are put into at least one cluster, was assessed for the cluster set produced by each method.

### Speed

The methods were also assessed by speed. As ISA and memISA are dependent on parallelisation to run at a reasonable speed, this is taken as real-world time taken to run, rather than computer run-time used. For k-means and penalised k-means, this includes the time taken to estimate k.

### GO enrichment

GO enrichment is a method that assesses the percentage of clusters that are significantly enriched (compared to all annotated genes on the microarray) with genes from one or more Gene Ontology categories (from the goa_human database) at different significance levels, using Fisher's exact test and the Benjamini false discovery rate multiple testing correction[23]. Clusters were tested for enrichment (using Fisher's exact test) for all GO biological process terms 3 or more levels deep into the hierarchical tree of GO terms, at several different levels of significance. At least 3 genes from the input cluster had to match a GO category for the cluster to be counted as enriched for that category, to ensure that chance appearance of 1 or 2 genes from a GO category with few members could not affect the results. The percentage of clusters matching this criterion gives a measure of the biological, functional relevance of the clusters.

GO enrichment was determined with the web-based service, GOstat[24]. This accepts multiple kinds of gene name or ID as input, allowing approximately 85% of genes within the input clusters to be included. This was automated using WWW-Mechanize, a Perl module[25].

To compare the results of GO enrichments for the various clustering algorithms, we also examined several random cluster sets using GO enrichment. Four sets of clusters with the same distribution of cluster sizes as those made by k-means (at the value of k recommended by cascadeKM), CRC, ISA and memISA (both after removal of overlapping clusters) were produced. The cluster sets made from the Dobrin, MC66 and PB datasets were combined when determining the distribution of sizes. The new cluster sets had genes chosen at random from all those available on the Affymetrix 133P chip.

### k-means

**Figure 1:**
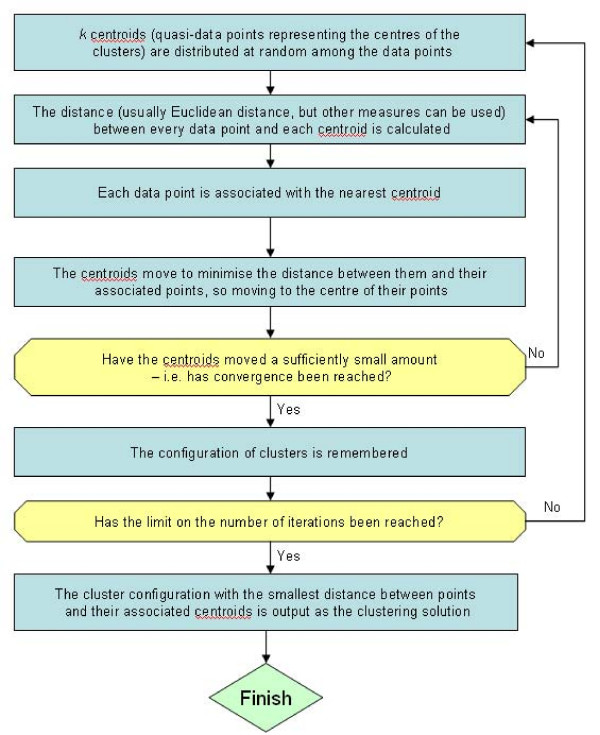
**Flowchart summarising the method used by k-means clustering**. k is a user-defined input parameter which sets the number of clusters k-means clustering will find.

k-means clustering is a standard clustering method that has been in use for several decades [26]. It requires that the user specify the number of clusters to sort the genes into (*k*). k-means clustering is a single cluster membership method – each gene can belong to only one cluster and it also assigns every gene to a cluster. Essentially, it distributes *k *centroids (quasi-data points representing cluster centres) throughout the data. Data points are then assigned to their nearest cluster, and the centroids are moved to minimise the distance between them and their assigned data points. This is repeated until the centroids stop moving. A number of distance measures can be used to define distance between data point and centroid, with Euclidean distance being one of the most commonly used and simplest. The procedure is summarised in Fig. 1.

There are numerous variants of k-means clustering [27,28]. Here, two are tried – standard k-means clustering, as above, and penalised k-means clustering. Penalised k-means clustering uses a threshold parameter (λ) to allow some of the genes to be treated as noise, and not clustered.

Initially, an estimate for the value of k was found for all three datasets using the cascadeKM function in R. Values of k between 2 and 35 were assessed, with 25 iterations per value, and the k values that minimised the Calinski criterion were chosen [29]. The recommended values of k were 6 for Dobrin, 7 for MC66 and 8 for PB cerebellum. k-means and penalised k-means were then performed on all four datasets at 200 iterations and these values of k. The recommended value of 0.1 was used for λ in penalised k-means.

These small values of k will only partition the data into several large clusters, which may be too general a grouping to provide biologically relevant inferences. To examine the performance of k-means when producing smaller, more specific clusters, and also for a more direct comparison to CRC, k-means and penalised k-means clustering were also performed with values of k equal to the numbers of clusters produced by CRC on that dataset (23 in all cases).

k-means was performed using Cluster 3.0 [10]. Penalised k-means clustering was performed using PWKmeans [28]. Both were performed on a Windows desktop PC with 2 GB RAM, using a 2.66 Ghz processor.

### CRC

CRC[11] is a model-based clustering method. The name arises from a metaphor where genes are regarded as customers in a Chinese restaurant with unlimited tables of unlimited size, each representing a cluster, and their food orders represent the expression profile of each gene. The customers are then seated at tables according to the similarities of their food orders. CRC has several advantages over other methods. It can handle missing data and cluster genes based on negative correlation and time-shifted correlation. Like k-means it is a single cluster membership method. Its methodology is complex, and is based upon treating the expression profiles of the genes as the sum of multiple normal distributions.

**Figure 2:**
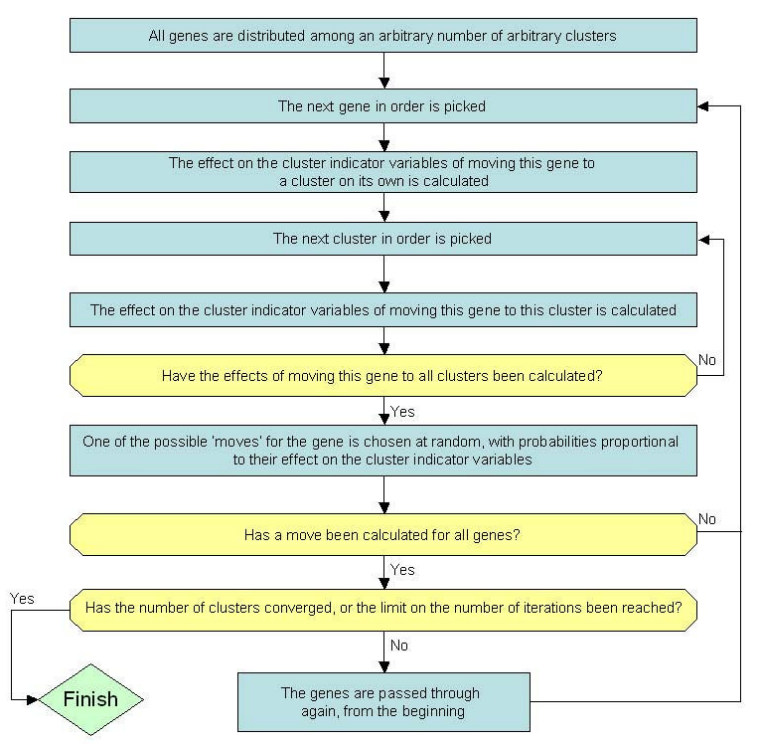
**Flowchart summarising the method used by CRC**. One run through this flowchart equates to a single chain in CRC, with several chains being run in parallel. The number of parallel chains and the maximum number of iterations are user-defined parameters.

The procedure is outlined in Fig. 2. Each iteration of the flowchart in Fig. 2 can be considered a Markov chain process. CRC runs a number of these chains in parallel (set by the user – 10 is the recommended amount), and reports the highest likelihood cluster set as the final output. The chains are also limited to a certain number of iterations through the flowchart before reporting their clusters. This is another parameter decided by the user, and is recommended to be set at 20. Finally, a probability cut-off can be input, which determines how high the likelihood of a gene being a member of a cluster needs to be in order for it to be included in the final output. In practice, most genes are members of their cluster with probability 1, so this removes few genes.

CRC was performed on all three datasets. It was performed at two parameter sets for each dataset – 10 chains/20 cycles per chain/probability cut-off of 0.7, and 20 chains/40 cycles per chain/probability cut-off of 0.9.

CRC was performed using a standalone program [11]. It was performed on a Unix server running Redhat OS with 32 GB RAM, using one 2.2 Ghz processor.

### ISA

**Figure 3:**
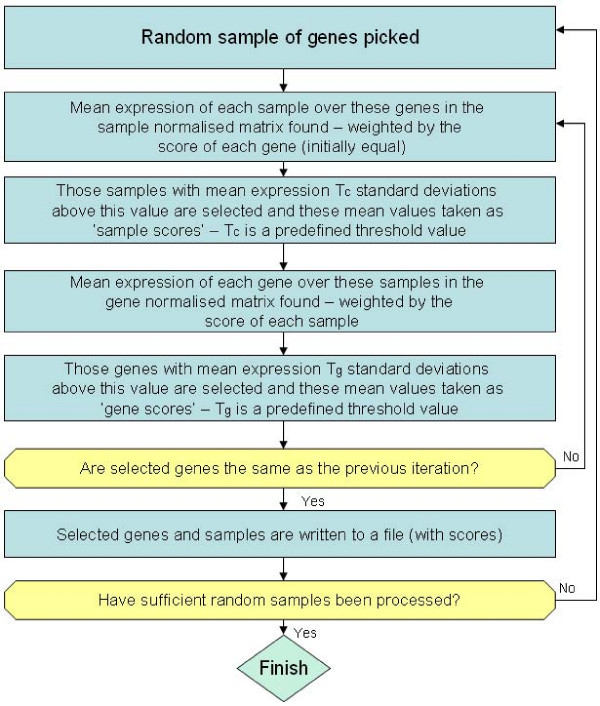
**Flowchart summarising the method used by ISA**. t_G _and t_C _are user-defined threshold parameters. They determine how great the level of expression for a gene or sample (defined in standard deviations from the weighted mean of all genes over those samples, or all samples over those genes) needs to be for selection in the cluster. Higher values lead to more, smaller clusters, lower values to fewer, larger clusters. A preliminary run at a low number of iterations, with a wide range of values for t_G _and t_C_, is used to determine a sensible range of t_G _and t_C _values for use in the main run.

ISA is a biclustering method – it clusters both rows and columns of the dataset, here the genes and the specific samples they come from [12,13]. This allows ISA to focus on subsets of samples with good signal for the genes of the cluster, reducing the amount of noise (see Fig. 3). Unlike k-means and CRC, it is not a single-cluster membership method: it allows genes and samples to belong to multiple clusters, and does not have to put every gene into a cluster. A high proportion of its clusters were found to be significantly enriched for one or more GO terms in yeast data[4].

ISA produces tens of thousands of clusters. In postprocessing, to reduce this set to a manageable size, duplicate clusters are removed, similar clusters are merged, and clusters can be reiterated through ISA. The nature of postprocessing affects the final clusters.

ISA also assigns 'scores' to genes and samples it has clustered, as part of its method. A gene or sample with a high score will have more influence on the samples or genes selected at the next stage of the process. The final values of these scores are reported in ISA's output. A high score here indicates that the gene or sample has had greater influence over the clusters' contents than a gene or sample with a low score.

ISA was used on the Dobrin datasets with 8 different postprocessing regimes (see Additional Files 2, ISAPostprocessing.doc, for details). The regime that produced the highest GO enrichments included filtering the clusters by size and number of occurrences, and using less stringent similarity criteria when combing similar clusters.

To compare with memISA, CRC and k-means, runs were performed on all three datasets, at 20000 iterations. These runs used t_G _values of 1.0 to 4.2 (inclusive, increasing in 0.1 intervals). Different t_C _values were used for different datasets, as each contained different numbers of samples – Dobrin was run at t_C _0.2, 0.5 and 1.0, MC66 at 0.25 and 1.25, and PB cerebellum at 0.1, 0.4 and 0.7. Filtering was used – a cluster had to have appeared 3 times, and contain at least 40 genes, to be included in the final output. Clusters that shared 70% or more of their genes with a larger cluster were removed from the final results (see below).

ISA was written in Perl (see Additional Files 3, ISAScripts.zip for a zip file containing all ISA and memISA scripts), based upon the previous Matlab implementation[13]. This implementation has all of the properties of the Matlab version. The postprocessing scripts were written in Perl. The normalisation script was written in R[20]. ISA was parallelised using Cardiff University's CONDOR network, which distributes individual ISA runs to unused Windows desktop computers across campus[30].

### memISA

The underlying method of memISA is closely based on ISA and similar to PISA[14] (Fig. 3). It biases against both genes and samples that have already been put into a cluster, according to two user input parameters, *f *and *n*. The bias is calculated relative to the highest scoring gene and sample in a cluster – this has its gene/sample score multiplied by the factor (1 - *f*) in future iterations. All other genes/samples found in a cluster have their future scores reduced by a smaller amount. This is determined by the proportion of their score and the highest gene/sample score – a gene with a quarter of the score of the highest gene will have its future scores multiplied by 1 - (f * 0.25). The intent of this is to bias against the highest scoring genes of a cluster while allowing lower scoring genes to be relatively unaffected and still be included in subsequent clusters (the highest scoring genes typically have scores 10 times greater than the majority of genes in a cluster). If a gene/sample is included in a subsequent cluster, the biases are multiplied together – a gene which is the strongest gene in two successive clusters would have its score multiplied by (1 - *f*)^2 ^in following iterations.

These biases are only remembered for a certain number of iterations (*n*). Every *n *iterations, the slate is wiped clean. This is to ensure that memISA does not begin returning noise once it has found all the available clusters in the data, and to limit the effect that an early misclustering can have on the results.

memISA was run on the Dobrin dataset at 20000 iterations with a number of different values for *f *and *n *(Table 2). It was found the results were generally robust to the values of *f *and *n*, and that *f *= 0.7 and *n *= 5 produced clusters with the highest GO enrichment, so these values were used in all further analysis. A filtering step was also attempted on one dataset to see if it would improve GO enrichment. For this, those genes whose gene scores were in the bottom 10% for their cluster were removed from the cluster. This step reduced both gene coverage and GO enrichment and so was not used further.

**Table 2:** Comparison of GO enrichments for different memISA parameters in Dobrin (overlaps not removed)

% enriched at varying p-vals	*f *= 0.5, *n *= 10	*f *= 0.75, *n *= 5	*f *= 0.75, *n *= 5, 10% of genes with lowest gene scores removed from clusters	*f *= 0.5, *n *= 3
p-val < 0.3	62.5	92.3	88.5	85.7

p-val < 0.1	50.0	65.4	57.7	60.7

p-val < 0.05	50.0	57.7	53.8	50.0

p-val < 0.01	43.8	42.3	42.3	46.4

p-val < 0.001	37.5	38.5	38.5	42.9

p-val < 0.0001	31.3	34.6	26.9	28.6

Gene coverage	61.1	78.8	74.7	74.7

Number of clusters found	16	26	26	28

memISA was run on the Dobrin, MC66 and PB cerebellum datasets at t_G _1.0 to 4.2 (inclusive, increasing in 0.1 intervals) and t_C _0.2, 0.5 and 1.0. Filtering was carried out as with ISA, using an occurrence threshold of 3 and a size threshold of 40.

memISA was implemented in Perl, and was based upon the new Perl implementation of ISA. Like ISA, it was parallelised using CONDOR.

### Assessing overlap between clusters

We examined inter-method overlap in gene membership of clusters for the four methods and intra-method overlap of ISA and memISA. CRC and k-means, as single-cluster membership methods, had no intra-method overlap between their clusters. ISA and memISA cluster sets, however, both contained a large amount of intra-method overlap, making them impossible to compare fairly with clusters produced by k-means or CRC. To try to facilitate fair comparison, clusters with gene overlap above a certain level (values of 60, 70 and 80% gene overlap were tried) were merged but since this resulted in datasets with fewer than 3 clusters, this approach was abandoned. As an alternative, where over 70% of the genes in the smaller of a pair of clusters was shared with a larger cluster, the smaller cluster was removed. This process was performed on a subset of ISA and memISA output – those raw clusters produced at t_G _= 2.1 or greater were used, and the rest discarded. This was in order to prevent a few very large clusters causing the removal of nearly all smaller clusters. This overlap removal step was applied after all other postprocessing.

### Combining methods

As there was not a large amount of overlap in clusters obtained between the ISA methods and either CRC or k-means, the possibility of combining their cluster sets to improve GO enrichment was investigated. The cluster sets were simply combined and clusters that had over 70% gene overlap with a larger cluster were removed as above. One set contained k-means, memISA and ISA clusters, one set contained CRC, memISA and ISA clusters. The memISA and ISA clusters had already had overlaps removed before combining. The CRC set used was the 10 chains/20 cycles per chain/0.7 cut-off. The k-means sets used were the k = 23 and k = 22 sets.

### Enrichment of clusters for schizophrenia related genes

The clusters produced from the combined k-means/ISA/memISA method on the Dobrin dataset were tested for enrichment with 607 genes associated with schizophrenia below a nominal threshold of p < 0.005 according to a recent genome-wide association study [31]using the program EASE [32], which implements a version of Fisher's Exact Test. Enriched clusters were also tested for enrichment for 352 genes differentially expressed between schizophrenics and controls in the analysis of the Stanley Medical Research Institute Online Genomics Database[16] at an uncorrected p-value of 0.02 or lower.

Clusters from combined k-means/ISA/memISA in the independent MC66 dataset that shared over 45% of their genes with any enriched cluster from the Dobrin dataset were then identified. Their enrichment for schizophrenia-associated genes and genes differentially expressed in schizophrenia was then determined with EASE. A permutation-based method of enrichment determination was also used. This allows the enrichment p-value for the MC66 clusters to be determined independently of the Dobrin cluster. 4000 pairs of clusters were constructed at random from the genes present on the Affymetrix 133A chip.

**Figure 4:**
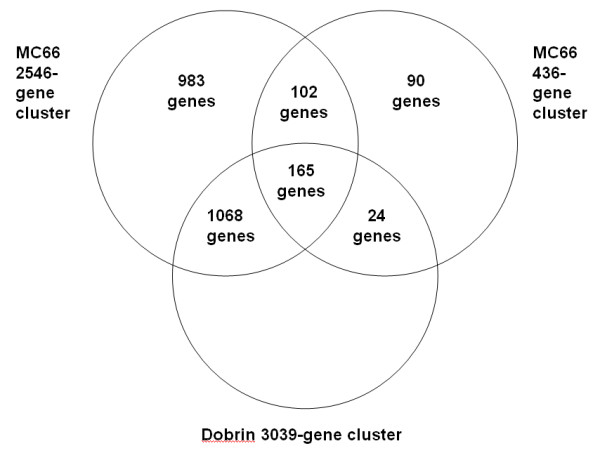
**Overlap between putative schizophrenia-related clusters produced from Dobrin and MC66 datasets**. Venn diagram showing the amount of overlap between the clusters enriched for schizophrenia-related genes, in order to construct randomised clusters for permutation.

The random clusters were constructed in pairs, as follows. Firstly, the number of genes shared between the three clusters was calculated (see Fig. 4). These figures were then used to create randomised MC66 clusters with the same level of overlap with the Dobrin cluster and each other.

165 genes from the Dobrin 3093-gene cluster were selected at random, and placed in both the 2546-gene and 436-gene MC66 randomised clusters. From the remaining Dobrin cluster genes, 1068 and 24 genes were selected at random, the former placed in the 2546-gene randomised cluster, the latter placed in the 436-gene randomised cluster. Then, 102 genes from the genes on the chip not present in the Dobrin 3093-gene cluster were selected at random, and placed in both the 2546-gene and 436-gene randomised clusters. From the remaining genes on the chip not present in the Dobrin 3093-gene cluster, 983 and 90 genes were selected at random, the former placed in the 2546-gene randomised cluster, the latter in the 436-gene randomised cluster. This was repeated 4000 times to produce a population of 8000 random clusters. These clusters were then processed with EASE in the same way as the original cluster, allowing the original results to be compared to them.

These clusters were also examined for enrichment in KEGG and BioCarta pathways, using the Composite Regulatory Signature Database [33] (), and for enrichment in GO biological process categories using GOstat.

EASE was also used to test these clusters for enrichment with genes found to be ten-fold or more upregulated in specific cell types within brain tissue according to Cahoy *et al *[34]-specifically, neurons, oligodendrocytes and astrocytes.

## Results and discussion

**Figure 5:**
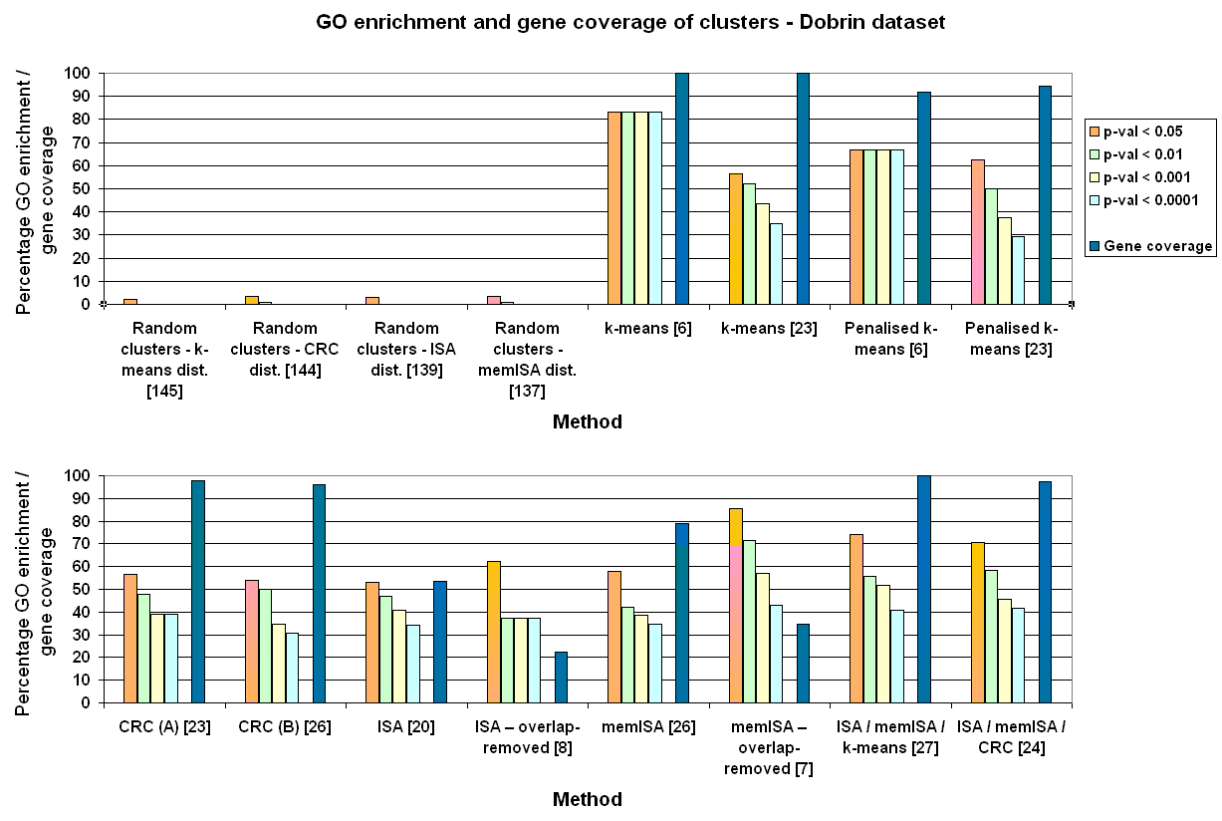
**GO enrichment and gene coverage of clusters for all methods – Dobrin dataset**. Orange, green, yellow and light blue bars are the percentage of clusters that are significantly enriched for one or more GO categories at p < 0.05, 0.01, 0.001 and 0.0001 respectively. Dark blue bar is gene coverage, the percentage of genes available on the chip that are assigned to at least one cluster. Numbers in square brackets are the number of clusters produced by that method. 'Dist.' = distribution of sizes. Parameter set A for CRC is 10 chains and 20 iterations per chain. Parameter set B for CRC is 20 chains and 40 iterations per chain.

**Figure 6:**
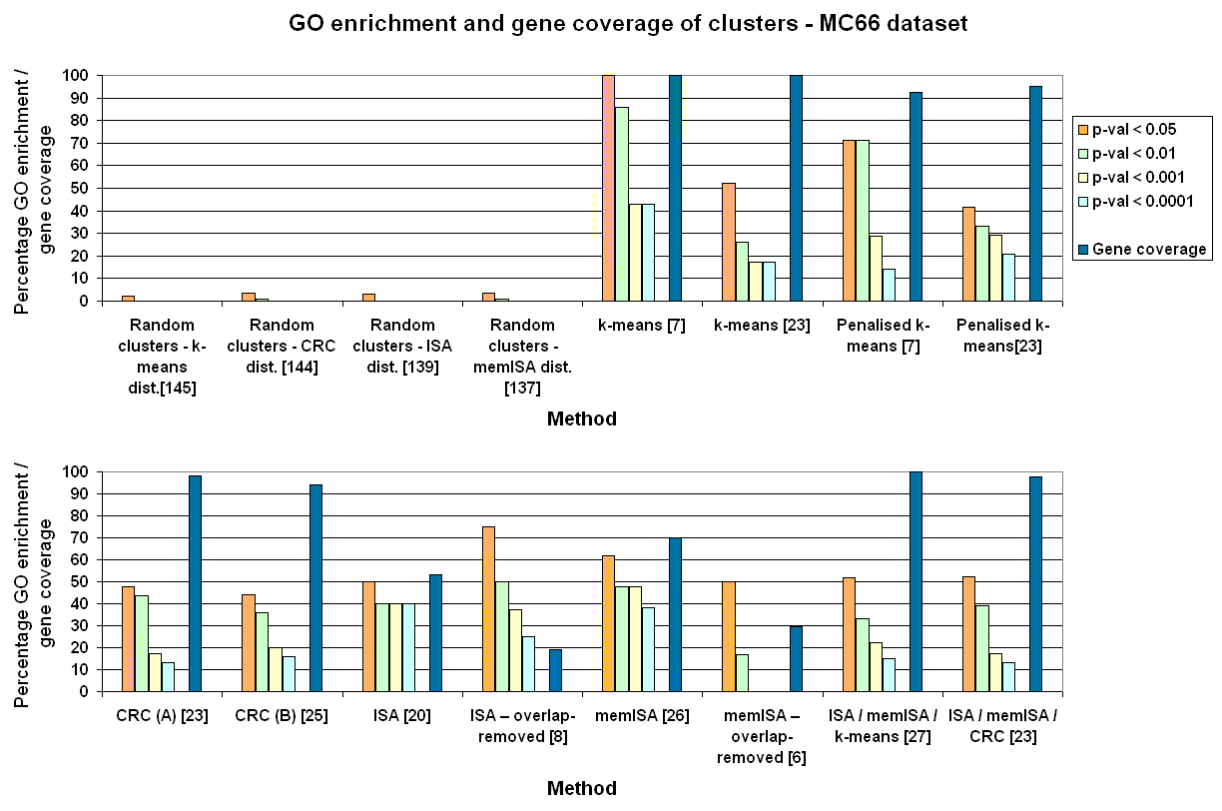
**GO enrichment and gene coverage of clusters for all methods – MC66 dataset**. Orange, green, yellow and light blue bars are the percentage of clusters that are significantly enriched for one or more GO categories at p < 0.05, 0.01, 0.001 and 0.0001 respectively. Dark blue bar is gene coverage, the percentage of genes available on the chip that are assigned to at least one cluster. Numbers in square brackets are the number of clusters produced by that method. 'Dist.' = distribution of sizes. Parameter set A for CRC is 10 chains and 20 iterations per chain. Parameter set B for CRC is 20 chains and 40 iterations per chain.

**Figure 7:**
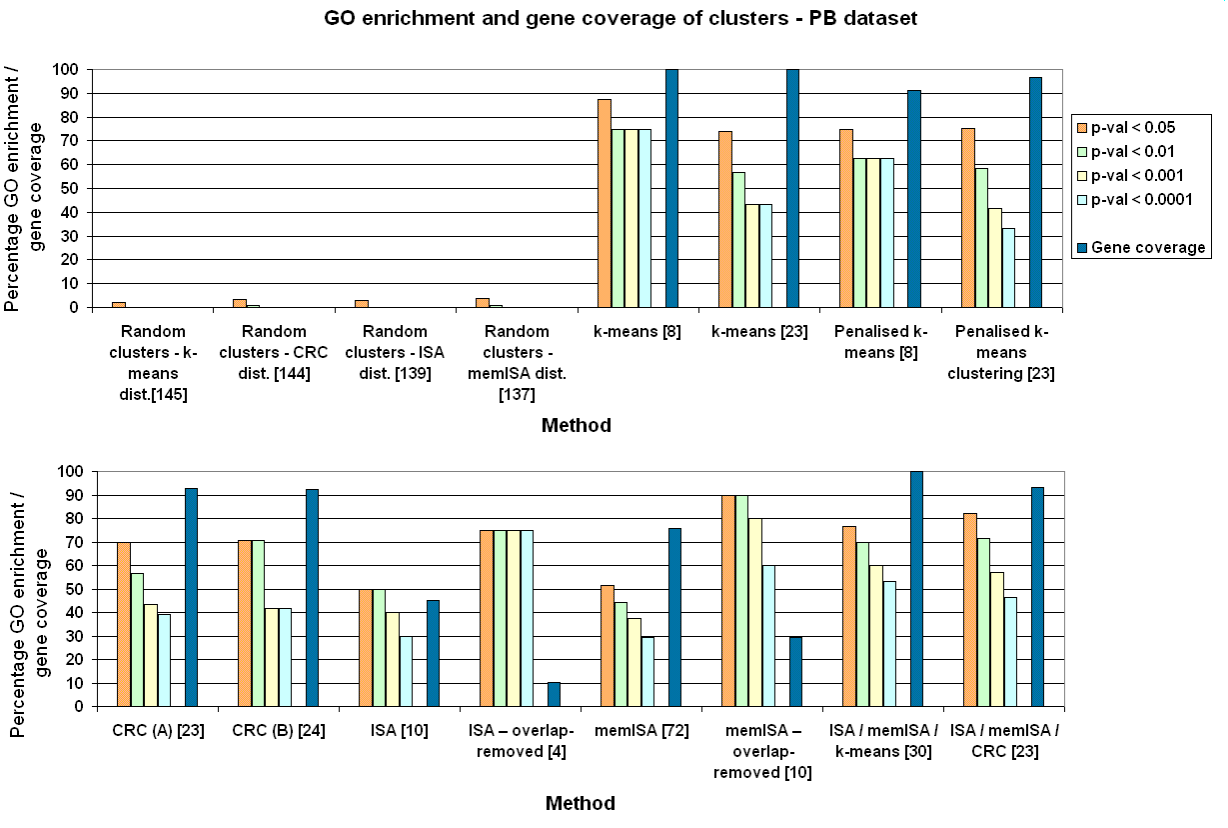
**GO enrichment and gene coverage of clusters for all methods – PB dataset**. Orange, green, yellow and light blue bars are the percentage of clusters that are significantly enriched for one or more GO categories at p < 0.05, 0.01, 0.001 and 0.0001 respectively. Dark blue bar is gene coverage, the percentage of genes available on the chip that are assigned to at least one cluster. Numbers in square brackets are the number of clusters produced by that method. 'Dist.' = distribution of sizes. Parameter set A for CRC is 10 chains and 20 iterations per chain. Parameter set B for CRC is 20 chains and 40 iterations per chain.

All four methods performed better than the random cluster sets when examined using GO enrichment to represent known biological relationships (Figs. 5, 6, 7). This implies that all the clustering methods result in groupings of biological significance. Of the three random cluster sets, those with the same size distribution as ISA had slightly lower GO enrichment than those with the same size distribution as memISA or CRC. This may suggest that GO enrichment has a small bias against ISA due to the sizes of clusters it produces. However, at p < 0.05 the difference dropped to under 1% GO enrichment, suggesting that any such bias is extremely slight and may well be due to chance.

### k-means and penalised k-means

k-means and penalised k-means produced clusters with high GO enrichments, especially at the lower k values recommended by cascadeKM. In these low-k cluster sets, k-means obtained higher GO enrichments than penalised k-means. In the k = 22 and k = 23 cluster sets, they produced cluster sets with similar GO enrichment (Figs. 5, 6, 7). As k-means gave similar GO enrichment to penalised k-means and by definition clustered more genes it was used in comparisons with the other methods.

### Effect of CRC parameters on GO enrichment

The different parameter sets used for CRC made little difference to the GO enrichments of its clusters. (Figs. 5, 6, 7). Increasing the numbers of iterations or cycles or increasing the probability cut off had little effect which suggests that altering these parameters is unnecessary, and that the default values of 10 cycles and 20 iterations per cycle should be used for most datasets, with parameters only being increased on very large datasets. One problem noted with CRC was that analysing more than 202 samples caused the program to crash. This occurred on both Windows and Linux versions of the program, so was presumed to be an inherent problem with the program.

### Effect of ISA parameters on GO enrichment

In contrast to CRC, changing the parameters of ISA can have unpredictable effects on the GO enrichment of its clusters, particularly after overlaps have been removed (see Figs. 5, 6, 7). The different values of t_C _used in memISA and ISA for the PB cerebellum and MC66 datasets may help explain some unexpected results – in particular, the very large number of clusters produced by memISA prior to removing the overlaps in PB cerebellum, and the unexpectedly poor performance of memISA on the MC66 dataset. However, these may also be due to chance differences in the selection of random starting clusters, or to inherent qualities of the methods.

### Effect of memISA parameters on GO enrichment

memISA is robust to the choice of *f *and *n*, as all of the combinations tried produced reasonable GO enrichments (see Table 2). *f *= 0.7 and *n *= 5 were chosen because they produced clusters with slightly better GO enrichments than other parameter sets.

### Comparison of clusters detected

There was a large amount of overlap between the clusters produced using penalised k-means and k-means at k = 23, with the majority of clusters (from all three datasets) having over 70% overlap with a cluster from the other method, and all others having over 40% overlap (see Table 3 and Additional Files 4 – AllOverlaps.xls for more detail). Since these methods found similar clusters, further analysis was focused on standard k-means clustering, as it had 100% gene coverage.

**Table 3:** Percentage overlap between clusters produced by different methods

	k-means	Penalised k-means	CRC	ISA	memISA
k-means	100	62.3	52.2	8.7	8.7

Penalised k-means	63.8	100	57.5	4.3	4.3

CRC	52.2	54.5	100	7.2	27.5

ISA	25	25	23.1	100	95.2

memISA	26.1	26.1	26.1	56.5	100

Values in table indicate the percentage of clusters produced by the method in the left margin that have over 70% gene overlap with one or more clusters produced by the method in the top margin.

There was considerable overlap in the results obtained between k-means and CRC across all three datasets. This suggests that k-means and CRC find similar patterns within the datasets. Conversely, there was little overlap between either k-means or CRC and either memISA or ISA clusters. In the case of ISA, there were a few overlaps at 70% or above for each dataset. In the case of memISA, there was a large cluster that overlapped with several of the smaller clusters produced by CRC at 70% or more, plus one other 70% plus overlap between more similarly sized clusters, in all three datasets.

Removing clusters with over 70% intra-method gene overlap from the ISA and memISA cluster sets reduced the number of clusters considerably. These sets contained only 4–10 clusters and were much smaller than the original ones. However, their GO enrichments were generally considerably higher (see Figs. 5, 6, 7) but at the cost of a considerable drop in gene coverage.

Nearly all ISA clusters had over 70% overlap with a memISA cluster across all three datasets. However, less than half of the memISA clusters had over 70% overlap with a cluster from ISA, as many of the ISA clusters overlap with the same memISA cluster. This level of overlap is surprisingly high, considering that their post-processing regimes already include a step to merge similar clusters. However, this step requires high sample overlap and correlation of shared gene/sample scores in addition to simple gene overlap. It also uses the size of the larger cluster to calculate overlap – i.e. 50% overlap in this step indicates that 50% of the genes in the larger cluster are found in the smaller cluster. As a result, it tends to only combine clusters of a similar size. The ability of memISA to bias against already-found clusters may help it find clusters that would previously have been hidden by a stronger cluster, a useful feature when looking for novel clusters.

The tendency of the cluster merging step in ISA and memISA to only combine clusters of a similar size may help to explain the improvement in GO enrichment the removal of overlapping clusters produces. Requiring a similar size and similar samples and gene/sample scores may help to ensure that only those clusters which come from the same signal are actually merged, excluding noise clusters with a coincidentally high gene overlap. The overlap removal process would then remove these clusters from the dataset altogether, improving GO enrichment.

The reasons for the poorer performance of memISA on the MC66 dataset are not known. It is possible that the difference in the t_C _and t_G _parameters between memISA and ISA for this dataset was critical. The smaller number of genes in this dataset might also be important, and so reducing the values of t_G _used may help. Alternatively, it might be that chance played a role. memISA may be inherently more prone to chance variation than ISA or CRC.

### Combining methods

The cluster sets produced by combining the methods had similar gene coverage to those produced by CRC/k-means alone (see Figs. 5, 6, 7). They generally had a higher number of clusters. For the CRC/ISA/memISA combined set, the GO enrichment of these clusters was higher in the Dobrin and PB cerebellum datasets,. In the k-means/ISA/memISA combined sets, the gains in GO enrichment relative to k-means alone were generally smaller: under 5% at most levels of p. There were a few small losses in GO enrichment in some datasets and at some levels of p, but generally the impact on GO enrichment was still positive.

### Gene coverage

Before highly overlapping clusters were removed from the clusters produced by ISA, k-means had the highest gene coverage (100% by definition), followed by CRC, and then by memISA and lastly ISA. However, these cluster sets are not directly comparable on number of clusters or on GO enrichment, as the cluster sets produced by ISA and memISA contain a large amount of redundancy.

As memISA and ISA had much lower gene coverage than k-means or CRC, the relationship between mean gene expression levels and cluster membership was examined for these methods in the Dobrin dataset. For both ISA and memISA, no significant correlation was found (r = -0.132 for ISA, r = -0.081 for memISA).

### Cluster size

The number of genes per cluster for each method and dataset was also examined, and the mean cluster size and standard deviation computed (see Additional Files 5, SizeDistribution.xls). Generally, CRC, k-means and penalised k-means were consistent in their cluster sizes, which appear to vary only with the number of genes in the dataset. The average cluster size was between 800 and 900 for these three methods in both 133P datasets (Dobrin and PB), and between 500 and 600 in the MC66 dataset. ISA generally produces clusters that are smaller than this, between 400 and 600 on average (with no obvious relationship to number of genes or samples in the dataset). memISA, conversely, is particularly prone to producing datasets with one or two particularly large clusters, giving it a higher average cluster size and standard deviation. This is because the larger number of unique clusters it produces makes it more likely for clusters to overlap and be merged, leading to these extremely large clusters.

To examine whether cluster size affected enrichment, cluster size was checked for correlation with log_10 _of the p-values of the best GO hit for each cluster (unenriched clusters were treated as having a p-value of 1). No significant correlation was found for any of the methods.

### Speed

The three datasets were used to evaluate approximate runtimes for the four methods (see Table 4). CRC and k-means are very fast methods, with a runtime of a few hours on current computer technology. ISA and memISA, meanwhile, are much slower, taking up to a month without parallelisation. Even with parallelisation using CONDOR, ISA and memISA can take over 24 hours for a full parameter set when post-processing is included. Restricting the parameters to t_G _2.1 and above, as in the non-overlapping cluster set before, reduces these times by up to half.

**Table 4:** Comparison of method runtimes

Runtime on different datasets	ISA (using CONDOR)	memISA (using CONDOR)	CRC – 10/20	CRC – 20/40
Dobrin	23 h 6 min	37 h 22 min	2 h 12 min	7 h 53 min

MC66	17 h 23 min	28 h 55 min	1 h 15 min	4 h 33 min

PB cerebellum	15 h 11 min	24 h 13 min	1 h 7 min	3 h 53 min

Table showing the real-world time taken for the methods to run on each dataset.

### Enrichment of clusters for schizophrenia related genes

The clusters produced from the combined k-means/ISA/memISA method on the Dobrin dataset were tested for enrichment with 607 genes associated with schizophrenia according to a recent genome-wide association study[31], using the program EASE [32]. These 607 genes each contained at least one SNP associated with schizophrenia at an Armitage p-value of 0.005 or under. One cluster, containing 3093 genes and originally found by memISA, was enriched (p = 0.0104 after Bonferroni correction for 26 clusters).

This cluster was also tested for enrichment with 352 genes found to be differentially expressed between schizophrenics and controls in the analysis of the Stanley Medical Research Institute Online Genomics Database[16] at an uncorrected p-value of 0.02 or lower. The cluster was slightly enriched, at a p-value of 0.09.

Clusters from combined k-means/ISA/memISA in the independent MC66 dataset that shared over 45% of their genes with this enriched cluster were then identified. Two clusters were found (containing 2546 and 436 genes respectively), both of which were nominally enriched for both schizophrenia-associated genes (2546-gene cluster at p = 0.0127, 436-gene cluster at p = 0.0117) and genes differentially expressed in schizophrenia (2546-gene cluster at p = 0.0064, 436-gene cluster at p = 0.00047 – see Additional Files 6, Clusters.xls, for the gene symbols of the genes in these clusters). However, since these clusters have some overlap with the 3093-gene Dobrin cluster, this cannot be considered independent replication of the original cluster.

To avoid this confounding effect, their enrichment for schizophrenia-associated genes and genes differentially expressed in schizophrenia was determined using a permutation-based method. The 436-gene cluster remained significantly enriched for the schizophrenia associated genes, while the 2546-gene cluster showed some enrichment, but this was insufficient to be significant (permutation p = 0.169 for the 2546-gene cluster, permutation p = 0.0255 for the 436-gene cluster). However, both clusters were significantly enriched for genes differentially expressed in schizophrenia (permutation p = 0.0053 for the 2546-gene cluster, permutation p = 0.0005 for the 436-gene cluster).

These clusters were also examined for enrichment in KEGG and BioCarta pathways, using the Composite Regulatory Signature Database [33]  (). The top hit for the Dobrin cluster and the 2546-gene MC66 cluster was the KEGG entry for the MAPK signalling pathway (p = 1.12e^-7^, FDR q = 0.00024 in Dobrin, p = 6.95e^-10^, FDR q = 1.46e^-6 ^in MC66). The only significant hit for the MC66 436-gene cluster was from the BioCarta Synaptic Junction pathway (p = 3.88e^-5^, FDR q = 2.71e^-2^).

The MC66 436-gene cluster was also examined using GOstat, where the best hit was for GO:0007399 (nervous system development) GO category (p = 0.044 after FDR correction).

The three clusters were also tested for enrichment with genes found to be ten-fold or more upregulated in specific cell types within brain tissue according to Cahoy *et al *[34]-specifically, neurons, oligodendrocytes and astrocytes. All three clusters were found to be highly significantly enriched with genes upregulated in neurons (p = 2.5e^-21 ^in Dobrin, p = 1.55e^-16 ^in MC66, Bonferroni corrected). There was also enrichment for genes upregulated in oligodendrocytes (Dobrin p = 0.06, MC66 p = 2.4e^-4^, Bonferroni corrected) and astrocytes (Dobrin p = 5.13e^-22^, MC66 p = 2.26e^-10^, Bonferroni corrected).

Three overlapping clusters, enriched to varying degrees for either schizophrenia-associated genes or genes differentially expressed in schizophrenia, were found from the two independent dorsolateral prefrontal cortex datasets. The apparent excess of schizophrenia-associated genes in the 2546-gene MC66 cluster could be explained by its overlap with the Dobrin cluster. Thus, this cluster does not constitute independent evidence for schizophrenia-associated genes clustering together with respect to their expression levels. However, the 436-gene MC66 cluster remained significantly enriched when assessed by the permutation method. Both MC66 clusters did show significant over-representation for genes differentially expressed in schizophrenia, even after correction for the overlap with the Dobrin cluster. This demonstrates the ability of the methods to find potentially disease-related gene clusters that are replicable in multiple datasets.

The large size of two of the clusters makes inferences about individual genes difficult. However, both the larger clusters are enriched for genes present in the KEGG MAP kinase pathway, suggesting that this pathway may relate to the aetiology of schizophrenia. Members of this pathway have also been found to be differentially expressed between controls and schizophrenics in other brain regions [35]. In addition, when structural variants such as microdeletions occur in the genomes of schizophrenics, they are particularly likely to occur in the genes of the MAP kinase pathway [36].

The smaller cluster was also found to be near-significantly enriched for serine/threonine kinase genes (the class of kinases which MAP kinases belong to), and also for synaptic junction and neurological development genes. As this cluster is enriched for both schizophrenia associated genes and genes differentially expressed in schizophrenia, further investigation of the role of these pathways in schizophrenia aetiology may be useful.

However, the MAP kinase-related genes present in the two large clusters do not overlap with the schizophrenia associated gene set or the differentially expressed in schizophrenia gene set (they share no genes at all in either the MC66 or Dobrin cluster). This might suggest the MAP kinase function of the clusters may be incidental to their roles in schizophrenia aetiology. Further investigation with other functional analysis tools may allow more biological inferences from these clusters.

### Comparisons with other clustering method surveys

Our findings broadly agree with several other surveys of clustering methods (Figs. 5, 6, 7). Like Prelić *et al*, we find that ISA is an effective method that produces clusters with high GO enrichment [4], but our cluster sets generally do not have as high a proportion of GO enriched clusters as theirs. This is likely to be a consequence of the greater complexity of the input data.

Garge *et al *found k-means clustering effective [15] on a wide range of input datasets. This is echoed by the k-means cluster sets reported here, which have high GO enrichment and gene coverage scores. These scores were generally higher than CRC, the mixture modelling method examined here. This contrasts with the findings of Thalamuthu *et al*, who found that modelling methods were superior to k-means clustering [9]. This difference is again likely to be due to the datasets used; in particular the datasets used here were much larger in size.

## Conclusion

k-means clustering, CRC, ISA and memISA are all potentially useful methods. Considered alone, k-means clustering is probably the most useful of the four, as it is fast, does not require parallelisation, and produces clusters with slightly higher levels of GO enrichment than CRC when producing similar numbers of clusters. When used to find smaller numbers of clusters more in line with the estimation of k, the GO enrichments are higher still, reaching 100% at some levels of p. It also assigns a cluster to every gene (100% gene coverage), unlike overlap-removed ISA and memISA (under 30% gene coverage). Although this must lead to some false positives, this does not seem to have affected the GO enrichment scores unduly, and is an advantage in exploratory studies where as wide a view as possible is desired. Furthermore, k-means is a relatively simple and very well understood method. This simplicity may be the reason for its good performance here, as it may allow it to cope with a wide variety of input data. CRC, conversely, has many more parameters and so may have had scope to become optimised for the smaller yeast and bacterial datasets it was built for and tested upon.

However, for the fullest picture of clusters available in a dataset, combining memISA, ISA and k-means is the best option, as it offers higher GO enrichment than k-means alone in two out of the three test datasets while retaining 100% gene coverage (see Figs. 5, 6, 7). Even in the MC66 dataset, it added additional clusters not found by k-means without reducing GO enrichment. One of these memISA clusters (found in both dorsolateral prefrontal cortex datasets) was found to be significantly enriched for schizophrenia-associated genes and genes differentially expressed in schizophrenia, further emphasising the utility of combining methods. If time allows, this combined method should be the method of choice for clustering microarray brain expression data.

## Authors' contributions

ALR wrote all programs, performed analyses and wrote the paper. PH designed the permutation method used to assess cluster enrichment. LJ, PH and MCO constructively evaluated and edited the paper, and advised additional analyses. MJO provided access to the schizophrenia association data.

## Supplementary Material

Additional file 1 **Survey of microarray expression data clustering methods**. Table showing the methods surveyed in the literature to decide which ones to investigate more closely. Click here for file

Additional file 2 **Details of ISA postprocessing regimes.** Table and description of ISA postprocessing regimes tried on the Dobrin dataset. Click here for file

Additional file 3 **Scripts to run ISA and memISA**. ZIP archive containing the Perl and R scripts needed to run the version of ISA and memISA described here. Includes Instructions.txt, a step-by-step guide to using them. Click here for file

Additional file 4 **Inter-method gene overlap**. Spreadsheet showing inter-method gene overlap for clusters from all methods, in all datasets. Overlap is defined as the percentage of genes present in the smaller cluster that are also found in the larger cluster. Click here for file

Additional file 5 **Distribution of cluster sizes**. Spreadsheet showing number of genes present (cluster size) in each cluster for each method across all datasets. Also shows mean cluster size and standard deviation of cluster sizes. Click here for file

Additional file 6 **Clusters enriched for schizophrenia-related genes**. Spreadsheet showing the three clusters described in the paper. The 3093-gene cluster was made from the Dobrin dataset by memISA, and the 2546-gene and 436-gene clusters were made from the MC66 dataset by memISA. Click here for file
