# Supplementary material for: A comparison of four clustering methods for brain expression microarray data
Source: BMC Bioinformatics. 2008 Nov 25;9:490. doi: 10.1186/1471-2105-9-490 (PMC2655095; doi:10.1186/1471-2105-9-490)
Supplement: Additional file 2 — Details of ISA postprocessing regimes. Table and description of ISA postprocessing regimes tried on the Dobrin dataset. [file 1471-2105-9-490-S2.doc]

ISA / memISA postprocessing

ISA was performed on the Dobrin dataset across a range of parameters and post-processing regimes in order to find the best combination. A preliminary run at a low number of iterations and a wide range of values of t_C_ and t_G_ (see Figure 3 for an explanation of these two parameters) was performed to find sensible starting values for t_C_ and t_G_. Initially, ISA was run using t_C_ values of 0.25 and 1.25 and t_G_ values between 1.0 and 4.7 (inclusive, increasing in 0.1 intervals), with 10000 iterations.

The effects of 3 post-processing options, each with 2 choices, on the clusters produced from the Dobrin dataset were investigated:

1) The presence or absence of filtering. With filtering used, low-occurrence clusters were removed – those that appear less than 3 times within those clusters produced by a single pair of parameters. Only completely identical clusters count towards this threshold. Also, filtering removed clusters containing less than 40 genes.

2) Reiteration, or not, of the resulting cluster sets through ISA.

3) When the clusters created with different parameter levels were combined, either stringent criteria (80% gene overlap, 70% sample overlap, Pearson correlation > 0.8 between the scores of shared genes and samples) or lax criteria (60% gene overlap, 50% sample overlap, Pearson correlation > 0.6 between the scores of shared genes and samples) were used. The 8 regimes given by different combinations of these 3 options were compared (see table below). The filtered, non-reiterated, lax combination criteria set was used in all further ISA and memISA analyses.

Effects of different post-processing techniques on GO enrichment of clusters derived from ISA of Dobrin dataset

|  | 1 | 2 | 3 | 4 | 5 | 6 | 7 | 8 |
| --- | --- | --- | --- | --- | --- | --- | --- | --- |
| Filtered for size and occurrence | No | No | No | No | Yes | Yes | Yes | Yes |
| Reiterated | Yes | Yes | No | No | Yes | Yes | No | No |
| Stringent combining criteria | No | Yes | No | Yes | No | Yes | No | Yes |
| % enriched at p-val < 0.3 | 78.2 | 80.0 | 80.2 | 82.3 | 86.2 | 84.2 | 75.7 | 87.0 |
| % enriched at p-val < 0.1 | 48.7 | 47.2 | 51.6 | 49.3 | 52.3 | 56.3 | 48.6 | 57.5 |
| % enriched at p-val < 0.05 | 34.6 | 36.4 | 38.5 | 39.6 | 40.0 | 46.2 | 45.9 | 48.7 |
| % enriched at p-val < 0.01 | 28.2 | 25.4 | 28.6 | 31.2 | 36.9 | 33.0 | 35.1 | 36.0 |
| % enriched at p-val < 0.001 | 23.1 | 17.1 | 25.3 | 22.6 | 30.8 | 23.7 | 29.7 | 24.9 |
| % enriched at p-val < 0.0001 | 20.5 | 12.5 | 18.7 | 17.9 | 27.7 | 19.2 | 27.0 | 19.9 |
